# Supplementary material for: The interactome of intact mitochondria by cross-linking mass spectrometry provides evidence for coexisting respiratory supercomplexes
Source: Mol Cell Proteomics. 2017 Dec 8;17(2):216–32. doi: 10.1074/mcp.RA117.000470 (PMC5795388; doi:10.1074/mcp.RA117.000470)
Supplement: Supplemental Data [file supp_RA117.000470_133922_0_supp_23978_fzffwf.zip › spectra_annotation/mito_DR_spectra_annotation/99-1-8-1-12-1.pdf]

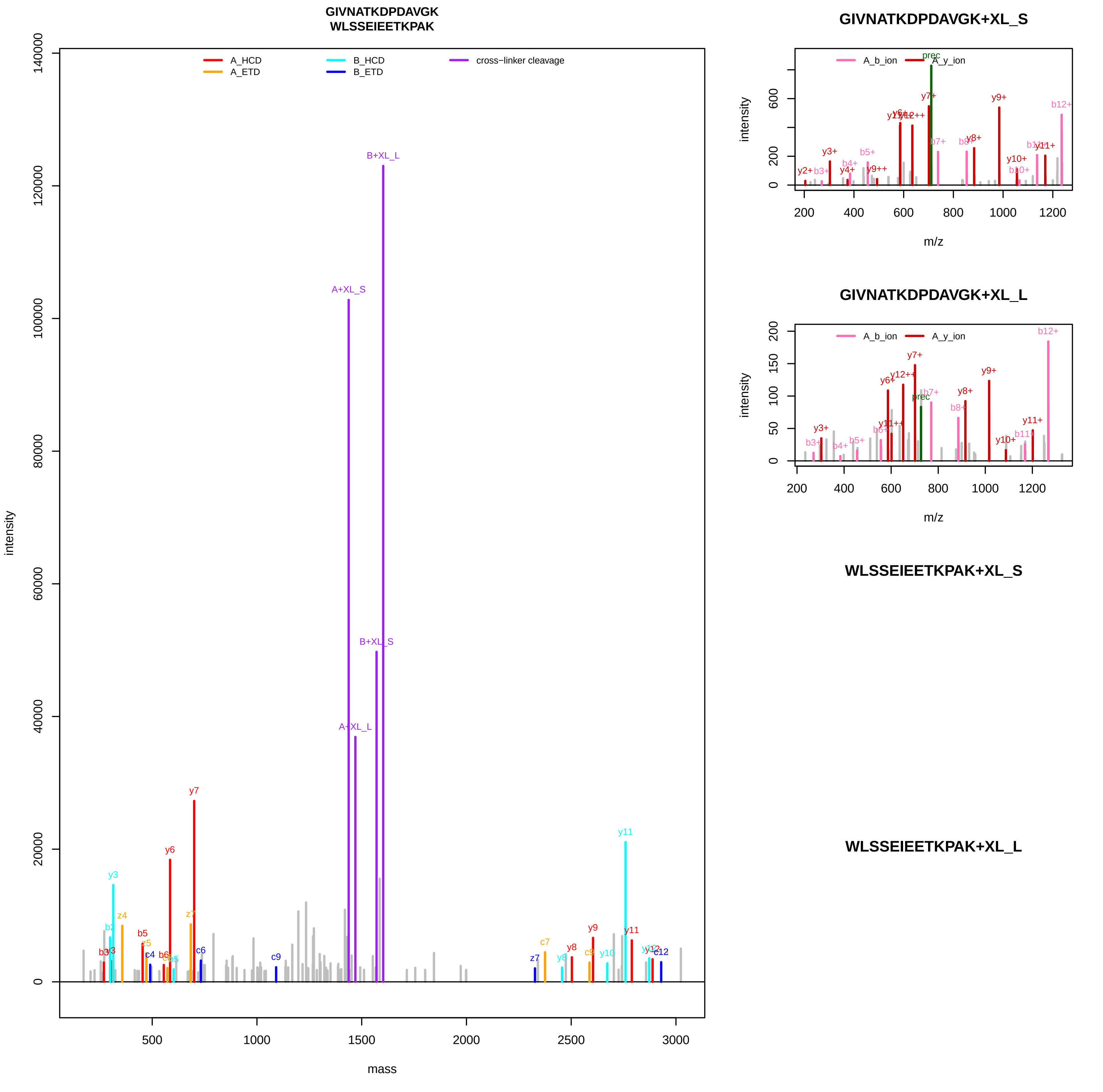
GIVNATKDPDAVGK
WLSSEIEETKPAK
A_HCD
A_ETD
B_HCD
B_ETD
cross−linker cleavage
B+XL_L
A+XL_S
B+XL_S
A+XL_L
intensity
mass
0
20000
40000
60000
80000
100000
120000
140000
500
1000
1500
2000
2500
3000
y7
y6
y3
b2
z4
b5
z5
c4
b6
c6
c9
z7
c7
y8
y9
c9
y10
y11
y12
c12
GIVNATKDPDAVGK+XL_S
A_b_ion
A_y_ion
prec
y7+
y9+
b12+
y6+
y11++
y12++
b7+
b8+
y8+
y3+
b5+
b4+
y4+
y9++
y2+
b3+
y10+
b10+
b11+
y11+
m/z
GIVNATKDPDAVGK+XL_L
WLSSEIEETKPAK+XL_S
WLSSEIEETKPAK+XL_L
